# Peripheral Branch Radiofrequency Thermocoagulation for Primary V2 Trigeminal Neuralgia: A Retrospective Cohort Study Guided by Diagnostic Nerve Blocks

**DOI:** 10.1155/prm/4938776

**Published:** 2026-07-29

**Authors:** Xi Dong, Chenyu Ding, Gaojian Tao

**Affiliations:** ^1^ Department of Pain, The Nanjing Drum Tower Hospital Clinical College of Xuzhou Medical University, Nanjing, Jiangsu, China; ^2^ College of Pharmaceutical Science, Zhejiang Chinese Medical University, Hangzhou, Zhejiang, China, zcmu.edu.cn

**Keywords:** infraorbital nerve, maxillary nerve, minimally invasive intervention, primary trigeminal neuralgia, radiofrequency thermocoagulation, recurrence

## Abstract

**Objective:**

To evaluate the clinical outcomes and safety of two peripheral branch radiofrequency thermocoagulation techniques—infraorbital nerve radiofrequency thermocoagulation (ION‐RFT) and maxillary nerve radiofrequency thermocoagulation (MN‐RFT)—guided by diagnostic block responses in a retrospective cohort of patients with primary V2 trigeminal neuralgia (TN), and to assess perioperative outcomes, including operative time, hospital stay, and healthcare costs, within this treatment pathway.

**Methods:**

This retrospective study included 92 patients with primary V2 TN treated at Nanjing Drum Tower Hospital between April 2021 and April 2024. All diagnostic nerve blocks were performed in the outpatient setting prior to hospital admission. Based on the diagnostic block responses, patients were divided into two groups: those who responded positively to the infraorbital nerve block received ION‐RFT, *n* = 45, and those who did not respond to the infraorbital block but subsequently responded to the maxillary nerve block received MN‐RFT, *n* = 47. Pain intensity was assessed using the visual analog scale (VAS) at baseline and at 1, 3, 6, and 12 months posttreatment. Recurrence rates were recorded at the same follow‐up intervals. Operative time was extracted from surgical records. Hospital stay, total healthcare costs, and complications were evaluated at the time of discharge.

**Results:**

Both treatment modalities resulted in significant postoperative pain reduction compared with baseline (within‐group effect, both *p* < 0.001). The incidence of postoperative complications and recurrence rates at all follow‐up time points did not differ significantly between the two groups (all *p* > 0.05). In terms of perioperative metrics, patients undergoing ION‐RFT had significantly shorter operative time (30.020 ± 11.957 min vs. 35.660 ± 10.602 min, *p* = 0.019) and shorter hospital stay (2.130 ± 1.200 days vs. 2.790 ± 1.610 days, *p* = 0.030) compared with those undergoing MN‐RFT, while total healthcare costs did not differ significantly between the two groups (5086.770 ± 1246.540 Yuan vs. 4988.250 ± 1144.350 Yuan, *p* = 0.694). Regarding pain outcomes, VAS scores in the ION‐RFT group showed a gradual increase from the immediate postoperative period to the 6‐month follow‐up, while scores in the MN‐RFT group remained relatively stable during this period (approach × time interaction, *F* = 4.000, *p* = 0.019); however, the difference in VAS scores between the two groups at 6 months did not reach statistical significance (*p* = 0.057).

**Conclusion:**

In patients with primary V2 TN, diagnostic block‐guided selection of ION‐RFT or MN‐RFT is a safe and viable treatment. Both techniques offer comparable pain relief, complications, and recurrence rates. ION‐RFT provides shorter operative time and hospital stay, reflecting faster recovery, while healthcare costs are similar between groups. These findings support a diagnostic block‐guided strategy that enables appropriately selected patients to receive a less invasive procedure without compromising efficacy.

## 1. Introduction

Trigeminal neuralgia (TN) is a debilitating neuropathic pain disorder characterized by sudden, severe, and recurrent facial pain within the sensory distribution of the trigeminal nerve [1–5]. Episodes are typically described as electric shock‐like and triggered by innocuous stimuli such as chewing or light touch [6–8]. TN most commonly affects the maxillary (V2) or mandibular (V3) divisions and is associated with significant psychological distress and impaired quality of life [9–12]. The International Classification of Orofacial Pain (ICOP) categorizes TN into primary and secondary forms [2, 13, 14].

Pharmacological therapy remains the cornerstone of initial management, with carbamazepine and oxcarbazepine as first‐line agents [2, 15, 16]. However, long‐term use is often limited by declining efficacy or adverse effects [9]. For refractory TN, interventional procedures become necessary. Microvascular decompression (MVD) is the gold standard for classical TN [17] but requires craniotomy and carries perioperative risks, limiting its suitability for elderly or comorbid patients [9, 18]. Consequently, less invasive percutaneous techniques such as radiofrequency thermocoagulation (RFT) have gained preference [19]. RFT enables selective lesioning of pain‐transmitting fibers and can be applied at the Gasserian ganglion or peripheral branches [9, 18, 20, 21]. Peripheral branch RFT may offer comparable efficacy to ganglion RFT while reducing complications such as corneal reflex loss or motor dysfunction [22–24].

For primary V2 TN, peripheral targets including the infraorbital nerve (ION) and the maxillary nerve (MN) are accessible via image‐guided approaches [9, 18, 19]. The MN provides sensory innervation to the midface, including the cheek, upper lip, and maxillary teeth [20, 21]. ION‐RFT targets the terminal branch and offers a straightforward trajectory with a favorable safety profile [9, 25, 26]. MN‐RFT via the foramen rotundum enables more proximal lesioning, potentially benefiting diffuse pain patterns, but involves greater procedural complexity [19, 27].

In clinical practice, the choice between ION‐RFT and MN‐RFT is guided by pain distribution and confirmed by diagnostic nerve blocks. Patients with pain localized to the infraorbital region typically undergo ION‐RFT following a positive infraorbital block. If ineffective, a maxillary block is performed; a positive response leads to MN‐RFT. If both blocks are ineffective, alternative strategies are considered.

Based on this clinical pathway, the present retrospective study compares the clinical efficacy, safety, and treatment‐related costs between patients with primary V2 TN who underwent ION‐RFT and those who received MN‐RFT. The objective is to elucidate the respective advantages and limitations of these two techniques and provide a clinical reference for optimizing interventional strategies for primary V2 TN.

## 2. Methods

### 2.1. Study Design and Patient Selection

**FIGURE 1:**
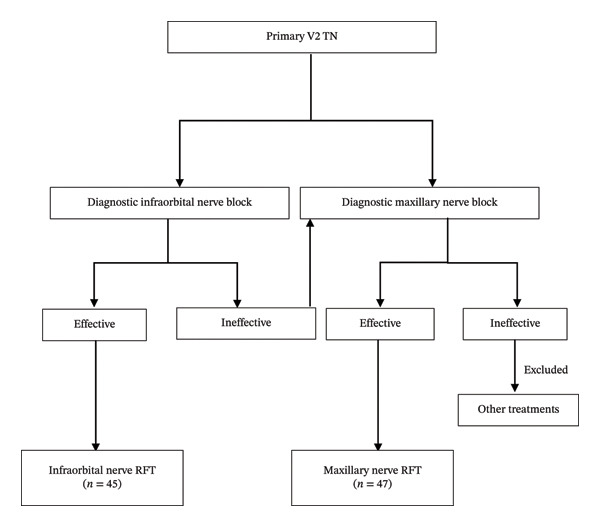
Study flowchart of patient enrollment and treatment allocation. Patients with primary V2 trigeminal neuralgia underwent diagnostic nerve blocks in the outpatient setting prior to hospital admission. Those who responded positively to the infraorbital nerve block (*n* = 45) received CT‐guided infraorbital nerve radiofrequency thermocoagulation (ION‐RFT). Patients who did not respond to the infraorbital nerve block but subsequently responded to the maxillary nerve block (*n* = 47) received CT‐guided maxillary nerve radiofrequency thermocoagulation (MN‐RFT). Patients with negative responses to both diagnostic blocks were excluded from the study and referred for alternative interventions, such as trigeminal ganglion RFT or trigeminal balloon compression.

The retrospective observational study was conducted at the Department of Pain Medicine, Nanjing Drum Tower Hospital, and approved by the Institutional Ethics Committee (Approval No. 2025‐0360‐01). In this study, a waiver of informed consent was applied for and granted, because this study retrospectively analyzed de‐identified clinical data from routine practice (including diagnostic nerve blocks performed in the outpatient setting and subsequent interventional treatments), with no research‐driven additional procedures or direct patient contact, and the study posed minimal risk to subjects. A total of 92 patients diagnosed with primary TN (both idiopathic and classical forms) involving the maxillary division (V2) of the trigeminal nerve was included between April 2021 and April 2024. All patients were evaluated and treated by a multidisciplinary pain management team and provided written informed consent prior to clinical procedures. The consent covered retrospective data use and procedure‐related documentation for CT imaging and blocks. Patients were assigned to two groups based on the site of RFT: the ION‐RFT Group (Group I, *n* = 45) and the MN‐RFT Group (Group M, *n* = 47). Assignment was determined by the site of effective diagnostic nerve block performed prior to RFT intervention. All patients received an ultrasound‐guided diagnostic block to confirm the affected nerve distribution. Patients demonstrating effective pain relief following ION block were assigned to Group I. Those with an inadequate response to the ION block or presenting with broader midfacial pain underwent MN block and were subsequently assigned to Group M (Figure 1).

Baseline demographic and clinical variables were recorded for each patient, including age, sex, laterality of symptoms, preoperative visual analog scale (VAS) pain scores, symptom duration, and the number of prior oral medication types.

Inclusion criteria include the following: (1) diagnosis of primary TN confined to the V2 distribution according to the International Classification of Headache Disorders (ICHD‐3); (2) Aae ≥ 40 years; (3) symptom duration ≥ 1 month; and (4) ability and willingness to undergo RFT and complete follow‐up assessments.

Definition of medication‐refractory TN: Patients were considered refractory to oral medication if they had failed at least two different classes of oral drugs (carbamazepine, oxcarbazepine, gabapentin, or pregabalin) taken at adequate therapeutic doses for a minimum of 1 month. Failure was defined as either (a) < 50% reduction in pain intensity on the VAS or (b) occurrence of intolerable adverse effects that led to discontinuation.

Exclusion criteria include the following: (1) secondary TN due to structural lesions (e.g., tumors and multiple sclerosis); (2) coagulation disorders or infection at the puncture site; (3) significant cognitive impairment or psychiatric illness that could interfere with pain reporting or compliance; and (4) incomplete clinical data or loss to follow‐up.

### 2.2. Preoperative Preparation and Diagnostic Block

For patients with primary TN affecting the second division, diagnostic nerve blocks were performed in the outpatient setting prior to hospital admission. All patients underwent preoperative diagnostic nerve blocks under ultrasound guidance to confirm the involvement of the target nerve. Since the ION is a branch of the MN, an ION block is first administered. If this is ineffective, an MN block is then carried out. Successful blockage is characterized by hypoalgesia (reduced sensitivity to pain) in the corresponding nerve distribution area of the patient and resolution of pain. Then, the appropriate surgical approach (ION or MN) is selected to perform the surgery.

The ION travels within the infraorbital canal and exits through the infraorbital foramen, requiring a relatively small volume of medication (0.5 mL of 2% lidocaine). In contrast, the MN runs within the pterygopalatine fossa and gives off numerous branches, thus necessitating a larger volume of medication (2 mL of 2% lidocaine). Preoperative blocks are typically performed in an outpatient setting or after hospital admission. The surgical procedure is conducted once the effect of the block has worn off. Therefore, intraoperative sensory function is still assessable.

**FIGURE 2:**
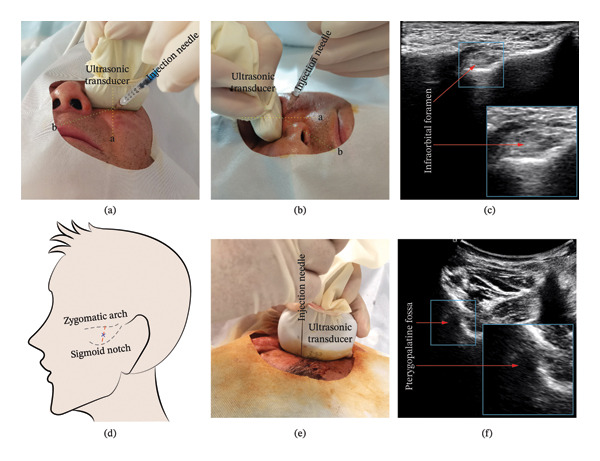
Diagnostic nerve blocks of the infraorbital nerve (ION) and maxillary nerve (MN) prior to CT‐guided radiofrequency thermocoagulation. (a)–(c) Infraorbital nerve block at the infraorbital foramen. (a) Ultrasound‐guided puncture of the infraorbital foramen, angled frontal view. (b) Frontal view photograph of infraorbital foramen puncture. (c) Representative ultrasound image showing the needle trajectory along the anterior maxillary surface into the infraorbital canal (inset: 2× magnified view of the infraorbital foramen). (d)–(f) Maxillary nerve block at the pterygopalatine fossa. (d) Schematic illustration of the puncture site for MN block. (e) Ultrasound‐guided puncture targeting the MN proximal to its exit from the foramen rotundum. (f) Representative ultrasound image of MN puncture (inset: 2× magnified view of the pterygopalatine fossa).

In Group I, the infraorbital foramen was localized at the intersection of a vertical line passing through the pupil (Figure 2a, b, line a) and the line connecting the outer canthus and midpoint of the upper lip (Figure 2a, b, line b). This landmark guided the needle trajectory for targeting the ION (Figure 2c). A diagnostic block is performed by the injection of 0.5 mL of 2% lidocaine. A positive response is defined as a rapid and significant reduction in spontaneous and evoked pain within 30 min, accompanied by a decrease in sensation at the site of the block, lasting for approximately 2 h. In Group M, the MN was accessed at the midpoint of the line connecting the zygomatic arch and the sigmoid notch (Figure 2d, blue X), with the needle advanced toward the pterygopalatine fossa under ultrasound guidance (Figure 2e, f). A diagnostic block was administered by injecting 2 mL of 2% lidocaine adjacent to the MN at the level of the foramen rotundum. A positive response is defined as a rapid and large reduction in spontaneous and evoked pain within 30 min, accompanied by a decrease in sensation at the site of the block, lasting approximately 2 h. Patients who did not achieve satisfactory analgesia with ION block were re‐evaluated and, if appropriate, underwent MN block. Only patients with a positive diagnostic response proceeded to therapeutic RFT. All procedures were performed in a dedicated interventional suite under strict aseptic conditions and continuous cardiac and respiratory monitoring.

### 2.3. RFT Procedure

**FIGURE 3:**
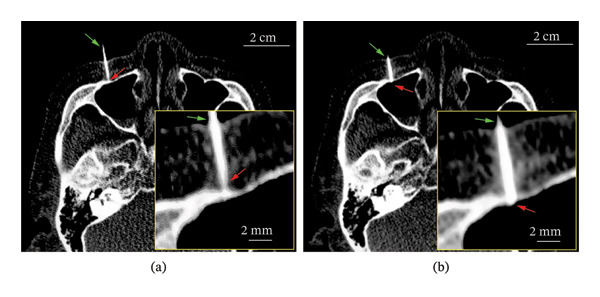
CT imaging of radiofrequency needle placement for infraorbital nerve targeting. (a) Axial CT scan showing the radiofrequency needle (green arrow) approaching the infraorbital foramen (red arrow), with the infraorbital canal and adjacent bony structures clearly visible. (b) Axial CT scan confirming the radiofrequency needle tip (green arrow) successfully inserted into the infraorbital canal. The red arrow shows proper intraforaminal needle placement. Insets display magnified views of the infraorbital foramen region in both panels for enhanced anatomical clarity and visualization of needle trajectory. These images demonstrate accurate needle guidance under CT for safe and effective infraorbital nerve radiofrequency thermocoagulation.

ION‐RFT (Group I): Patients were positioned supine with the head stabilized. After sterilizing the puncture site, local skin anesthesia was administered. Under CT guidance, a radiofrequency needle was advanced along the predetermined trajectory to the infraorbital foramen (Figure 3a, b). Needle position was confirmed by sensory stimulation at 50 Hz (0.3 V), eliciting pain in the ION distribution. Once confirmed, 0.5 mL of 2% lidocaine was injected for local anesthesia. RFT was then performed by gradually increasing the temperature to 80°C and maintaining it for 90s, repeating for 2‐3 cycles depending on patient tolerance. After lesioning, the needle was withdrawn, gentle compression was applied for hemostasis, and a sterile dressing was placed.

**FIGURE 4:**
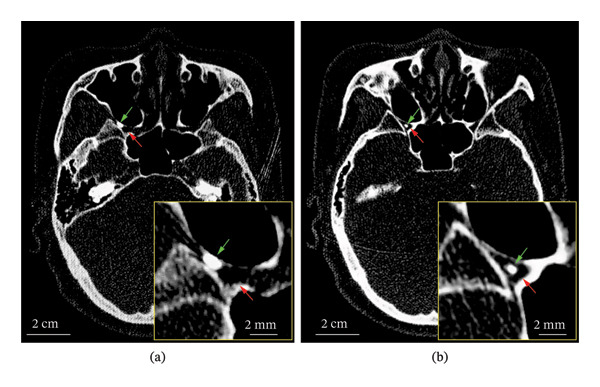
CT‐guided radiofrequency needle placement targeting the maxillary nerve via the foramen rotundum. (a) Axial CT image showing the radiofrequency needle (green arrow) approaching the foramen rotundum (red arrow). (b) Axial CT image confirming successful needle (green arrow) insertion into the foramen rotundum (red arrow), with the needle tip accurately positioned adjacent to the maxillary nerve. Insets provide magnified views of the foramen rotundum region, illustrating precise needle alignment and entry trajectory. These images validate the accuracy and safety of CT‐guided maxillary nerve radiofrequency thermocoagulation for the treatment of V2 trigeminal neuralgia.

MN‐RFT (Group M): Patients were positioned supine and prepped in the same manner as Group I. Under CT guidance, a radiofrequency needle was inserted perpendicular to the skin and advanced toward the foramen rotundum (Figure 4a, b). Upon contacting the lateral pterygoid plate, the needle was slightly withdrawn and redirected to reach the target site. Needle placement was confirmed by sensory stimulation at 50 Hz (0.3 V), reproducing pain in the MN distribution. Following confirmation, 0.5 mL of 2% lidocaine was injected for local anesthesia. Lesioning was then performed by gradually increasing the temperature to 80°C and maintaining it for 90s, repeating for 2‐3 cycles depending on patient tolerance. After completion, the needle was withdrawn, hemostasis was achieved with gentle compression, and standard postprocedural care was provided.

### 2.4. Postoperative Management

All patients were instructed to remain on bed rest following the procedure. Empiric antibiotic prophylaxis was administered to minimize the risk of postoperative infection. Throughout the hospital stay, patients were closely monitored for potential complications, including facial numbness, localized swelling at the puncture site, masticatory muscle weakness, and ocular irritation. Discharge was considered once patients demonstrated stable recovery, adequate pain control, and no evidence of serious adverse events. Many patients were discharged on the same day (surgery day). If patients experienced recurrent pain or developed an infection, they needed to remain hospitalized for observation and receive analgesic treatment.

Postoperative complications were managed according to standardized clinical protocols to minimize discomfort and prevent long‐term sequelae: (1) Facial numbness—Patients experiencing sensory loss in the affected facial region were instructed to avoid excessive rubbing, scratching, or applying heat or cold to the desensitized area and were advised to use protective measures such as facial coverings in extreme temperatures to prevent inadvertent injury due to impaired sensation. (2) Masticatory muscle weakness—A graduated rehabilitation plan was initiated for patients with reduced chewing function, starting with a soft or pureed diet and progressively transitioning to regular foods as muscle strength and coordination improved, supplemented by gentle daily chewing exercises to facilitate neuromuscular recovery. (3) Puncture site swelling or hematoma—localized swelling or hematoma at the puncture site within the first 24 h was managed with intermittent cold compresses (30 min on, every 3–5 min), followed by warm, moist compresses at 50°C–60°C after 24 h to promote circulation and hematoma resorption, with precautions taken to prevent thermal injury. (4) Keratitis or ocular irritation—Patients presenting with ocular dryness, irritation, or suspected keratitis were treated with sodium hyaluronate eye drops and referred promptly to an ophthalmologist for further evaluation and management, ensuring early intervention to prevent corneal complications.

### 2.5. Observation Indicators and Efficacy Assessment

This study compared a range of perioperative and postoperative clinical parameters between patients undergoing ION‐RFT (Group I) and MN‐RFT (Group M). Pain intensity was measured using the VAS, a 10‐cm horizontal line anchored at 0 (“no pain”) and 10 (“worst imaginable pain”), with patients marking the point corresponding to their perceived pain level. VAS assessments were performed preoperatively and at 1, 3, 6, and 12 months postoperatively. Additional parameters included the incidence of postoperative complications (facial numbness, masticatory muscle weakness, puncture site swelling, and keratitis), length of hospital stay and total healthcare costs. Pain recurrence was defined as the return of symptoms necessitating the resumption of pharmacologic therapy or repeat surgical intervention. Interventions were deemed ineffective if pain persisted without significant improvement, high VAS scores remained, or recurrence occurred during follow‐up. These measures enabled an evaluation of the clinical effectiveness and safety profiles of the two RFT techniques in managing primary V2 TN.

### 2.6. Statistical Analysis

All statistical analyses were performed using SPSS Version 26. 0. Categorical variables were expressed as percentages, with between‐group comparisons conducted using the chi‐square (*χ* ^2^) test. When *n* ≥ 40 and all cells had an expected frequency ≥ 5, the Pearson chi‐square test was applied; when *n* ≥ 40 and any cell had an expected frequency between 1 and 5, the continuity‐corrected chi‐square test was used; and when *n* < 40 or any cell had an expected frequency < 1, Fisher’s exact test was employed. Normally distributed continuous variables were expressed as mean ± standard deviation ($\overset{¯}{x}$ ± SD) and compared between groups using the independent‐samples *t*‐test, while within‐group comparisons were conducted using the paired‐samples *t*‐test. Nonnormally distributed continuous variables were reported as median (P25, P75), with between‐group comparisons performed using the Mann–Whitney U test and within‐group comparisons using the Wilcoxon signed‐rank test. Repeated measures ANOVA was used to test the multiple VAS score measurements postoperatively. Ordinal variables were analyzed using the rank‐sum test. A *p* value < 0.05 was considered statistically significant.

## 3. Results

### 3.1. Baseline Comparability Between Infraorbital and Maxillary RFT Groups

A total of 92 patients with primary TN involving the V2 division were included, comprising 45 patients in the ION‐RFT group (Group I) and 47 in the MN‐RFT group (Group M). No statistically significant differences were observed between the two groups with respect to age, sex distribution, side of pain, symptom duration, the number of prior oral medication types, or baseline VAS scores (*p* > 0.05), confirming that the groups were comparable at baseline (Table 1).

**TABLE 1:** Comparison of baseline characteristics between the two patient groups.

	Group I (*n* = 45)	Group M (*n* = 47)	*p*
Age (years)	72 (63, 82)	71 (65, 77)	0.705
Gender (male/female)	23/22	17/30	0.207
Preoperative VAS scores	5.710 ± 0.991	5.980 ± 1.073	0.201
Location (left/right)	26/19	23/24	0.538
Duration (month)	12 (4.5, 54)	24 (6.0, 96)	0.220
Number of prior oral medication types	2 (2, 3)	2 (2, 3)	0.778

*Note:* Continuous variables are presented as mean ± standard deviation or median (interquartile range) as appropriate. Preoperative and follow‐up VAS scores were compared using repeated‐measures ANOVA; Nonnormally distributed continuous variables (e.g., age, symptom duration, and number of prior oral medication types) were compared using the Mann–Whitney *U* test. Categorical variables (e.g., gender and laterality) were compared using the chi‐square test or Fisher’s exact test, as appropriate. A two‐sided *p* value < 0.05 was considered statistically significant.

The reasons for proceeding to interventional treatment were not mutually exclusive. In Group I, poor efficacy was present in 43 patients (95.6%), adverse effects in 9 (20.0%), and patient requests in 1 (2.2%). In Group M, the corresponding numbers were 45 (95.7%), 8 (17.0%), and 2 (4.3%). Percentages sum to > 100% because patients could have multiple reasons.

### 3.2. RFT Approaches Significantly Reduced Pain

Both treatment groups exhibited marked and sustained reductions in facial pain intensity, as measured by the VAS, following RFT. Based on repeated measures ANOVA of VAS pain scores from preoperative to 12 months postoperative, this study demonstrates no significant difference in baseline pain scores between the two groups (*p* = 0.201), ensuring comparability; both groups showed significant pain improvement at all postoperative time points compared to preoperative levels (within‐group effect *p* < 0.001). Although no statistical difference was found in overall efficacy between the approaches (main effect of approach *p* = 0.215), a significant “approach × time” interaction effect was observed (*p* = 0.019), indicating different pain relief trajectories. Specifically, Group M demonstrated a trend toward superiority over Group I at the mid‐term postoperative period (6 months, *p* = 0.057), with more durable and stable pain control, while Group I showed significant pain rebound at 6 months (*p* < 0.05 compared to immediate postoperative scores), and Group M maintained stable pain scores through the first 6 months. By 12 months postoperative, no significant difference existed between groups (*p* = 0.653), though both showed some pain recurrence compared to mid‐term scores. Therefore, we conclude that while both surgical approaches effectively relieve pain with equivalent long‐term outcomes, the data suggest that Group M may offer more stable pain control in the mid‐term (3–6 months) period, potentially contributing to a better quality of recovery during this critical phase (Table 2).

**TABLE 2:** Comparison of visual analog scale pain scores between two approaches over time.

Timepoint	Group I	Group M	*F*	*p*
Preoperative VAS	5.710 ± 0.991	5.980 ± 1.073	1.686	0.201
Postoperative VAS	0.600 ± 0.751^∗^	0.550 ± 0.619^∗^	0.019	0.890
1‐month VAS	0.910 ± 1.258^∗^	0.550 ± 0.620^∗^	2.157	0.149
3‐month VAS	1.130 ± 1.502^∗^	0.770 ± 1.088^∗^	2.391	0.129
6‐month VAS	1.840 ± 2.056^∗^	1.110 ± 1.550^∗^	3.825	0.057
12‐month VAS	2.710 ± 2.361^∗^	2.380 ± 2.516^∗^	0.205	0.653
Within‐Group Effect F	148.062	200.706		
Within‐Group Effect P	< 0.001	< 0.001		
Overall Test: Approach			*F* = 1.580	*p* = 0.215
Overall Test: Time			*F* = 176.432	*p* < 0.001
Overall Test: Approach × Time			*F* = 4.000	*p* = 0.019

*Note:* Data were analyzed by repeated‐measures ANOVA. All pairwise comparisons were adjusted using the Bonferroni correction. ^∗^ *p* < 0.05 vs. preoperative VAS. Within‐group comparisons at other time points are not shown in the table; all postoperative time points showed significant improvement from baseline (within‐group effect: *F* = 148.062 for Group I and 200.706 for Group M, both *p* < 0.001).

### 3.3. Similar Recurrence Rates Observed in Both Groups

Recurrence rates of trigeminal pain are the primary outcome of this research. Follow‐up data demonstrated an expected increase in pain recurrence rates over time in both groups. At the 1‐month follow‐up, the recurrence rate was 6.7% (3/45) in Group I compared to 0.0% (0/47) in Group M, a difference that was not statistically significant (*p* = 0.113). This trend continued throughout the observation period. By the 12‐month follow‐up, recurrence rates had risen to 44.4% (20/45) in Group I and 34.0% (16/47) in Group M. Although Group I exhibited a numerically higher recurrence rate at this final interval, the difference remained statistically nonsignificant (*p* = 0.393). The analysis of odds ratios (OR) further corroborated the lack of a significant efficacy difference between the two surgical approaches. The ORs for recurrence at 3, 6, and 12 months were 0.356 (95% CI: 0.065–1.935), 0.368 (95% CI: 0.117–1.162), and 0.645 (95% CI: 0.278–1.498), respectively. Recurrence was defined as the reappearance of facial pain necessitating either the reinitiation of pharmacologic therapy or a repeat interventional procedure. The majority of recurrences occurred between 6 and 12 months after treatment, and all affected patients reported a gradual increase in pain intensity rather than abrupt recurrence. Statistical comparison of recurrence rates revealed no significant difference between the two groups (*p* > 0.05), suggesting that the long‐term durability of pain relief was comparable regardless of the anatomical target. Notably, the recurrence rates in both cohorts remained within the range reported in previous studies of percutaneous radiofrequency interventions [25], supporting the overall efficacy of both approaches. These findings further indicate that peripheral RFT targeting either the ION or MN provides a similarly favorable long‐term outcome for patients with V2 TN (Table 3).

**TABLE 3:** Comparison of postoperative recurrence rates between two groups (*n*, %).

Time point	Group I (*n* = 45)	Group M (*n* = 47)	*p*	OR	95% CI
1 month after operation	3 (6.7)	0 (0.0)	0.113	‐^∗^	‐^∗^
3 months after operation	5 (11.1)	2 (4.2)	0.262	0.356	(0.065, 1.935)
6 months after operation	11 (24.4)	5 (10.6)	0.102	0.368	(0.117, 1.162)
12 months after operation	20 (44.4)	16 (34.0)	0.393	0.645	(0.278, 1.498)

*Note:* Intergroup comparisons were performed using the Chi‐square test or Fisher’s exact test, as appropriate. The odds ratio (OR) with a 95% confidence interval (CI) was calculated to quantify the association. A *p* value of < 0.05 was considered statistically significant.

^∗^OR and 95% CI were not calculated for the 1‐month follow‐up as no events occurred in Group M.

### 3.4. Similar Postoperative Complications

The postoperative complications observed in both groups are summarized as follows. Facial numbness was the most commonly reported adverse effect, occurring in 37 patients (16 mild versus 21 significant) in Group I and 43 patients (14 mild versus 29 significant) in Group M. Masticatory muscle weakness was observed in 2 patients in Group I and 5 in Group M; puncture site swelling was reported in 3 patients in Group I and 1 in Group M; keratitis was present in 2 patients in Group I and none in Group M. No significant differences were observed in complication rates between the two groups (*p* > 0.05) (Table 4).

**TABLE 4:** Comparison of complications between two groups (*n*, %).

Complications	Group I (*n* = 45)	Group M (*n* = 47)	*p*
Facial Numbness			0.226
Mild	16 (35.6)	14 (29.8)	
Significant	21 (46.7)	29 (61.7)	
Masticatory Muscle Weakness	2 (4.4)	5 (10.6)	0.435
Puncture Site Swelling	3 (6.6)	1 (2.1)	0.356
Keratitis^∗^	2 (4.4)	0 (0.0)	0.237

*Note:* Mild numbness refers to mild sensory disturbances in the facial skin without affecting daily life, with normal perception of touch and temperature. Significant numbness refers to pronounced facial numbness with hypoesthesia or sensory loss that affects daily life, with markedly reduced or absent perception of touch and temperature. Intergroup comparisons were performed using the chi‐square test or Fisher’s exact test, as appropriate. A *p* value of < 0.05 was considered statistically significant.

^∗^All patients with keratitis received emergency treatment, recovered well, and were left with no sequelae.

### 3.5. Shorter Operative Time and Hospital Stay With ION‐RFT

All patients received care from the same medical team using standardized protocols. Group I had significantly shorter operative time (30.02 ± 11.96 min vs. 35.660 ± 10.90 min, *p* = 0.019) and hospital stay (2.13 ± 1.20 days vs. 2.79 ± 1.61 days, *p* = 0.030) compared with Group M. Diagnostic nerve blocks were performed in the outpatient setting; thus, the difference in hospital stay reflects postoperative recovery rather than preprocedural evaluation. Total healthcare costs did not differ significantly between groups (5086.77 ± 1246.54 Yuan vs. 4988.25 ± 1144.35 Yuan, *p* = 0.694), indicating comparable cost‐effectiveness (Table 5).

**TABLE 5:** Comparison of hospital stay and costs between groups (x ± SD).

Outcome	Group I (*n* = 45)	Group M (*n* = 47)	t ∗∗	*p*
Length of Hospital Stay (days)	2.130 ± 1.200	2.790 ± 1.610	−2.198	0.030
Healthcare Costs (Yuan)	5086.770 ± 1246.540	4988.250 ± 1144.350	0.395	0.694
Duration of Surgery (minutes)	30.020 ± 11.957	35.660 ± 10.602	−2.395	0.019

*Note:* Data are presented as mean ± standard deviation. Independent samples t ∗∗‐test was used for between‐group comparisons.

## 4. Discussion

The treatment of primary V2 TN remains difficult, especially for patients unresponsive to or intolerant of medication [28]. MVD, though the standard surgical option, requires craniotomy and carries significant perioperative risks, limiting its use in elderly or frail patients [29, 30]. Additionally, not all patients have neurovascular compression [31]. In this context, percutaneous minimally invasive procedures such as RFT offer valuable alternatives. RFT exploits the differential heat sensitivity of myelinated fibers to selectively ablate pain‐conducting fibers while preserving tactile function [32–34]. The present study used parameters of 80°C for 90 s per cycle, with 2‐3 cycles [35]. While RFT has traditionally targeted the Gasserian ganglion [36, 37], advances in needle design and imaging guidance now allow lesioning of peripheral branches—such as the ION and MN—potentially reducing complication rates.

In this study, we evaluated the clinical efficacy of ION‐RFT and MN‐RFT, selected based on diagnostic block responses, for primary V2 TN. Over 12 months, both techniques significantly reduced pain intensity compared with baseline (both *p* < 0.001). The difference in VAS scores between the two groups at 6 months did not reach statistical significance (*p* = 0.057), although a significant approach × time interaction (*p* = 0.019) indicated differential temporal trends: VAS scores in the ION‐RFT group showed a gradual increase from the immediate postoperative period to the 6‐month follow‐up, while scores in the MN‐RFT group remained relatively stable during this period. This interaction has clinical implications. The more stable pain control with MN‐RFT suggests longer‐lasting relief, which may benefit patients with longer life expectancy or high recurrence risk. Conversely, despite some pain rebound by 6 months, ION‐RFT still significantly reduced pain and may be reasonable for older patients or those with shorter expected follow‐up. Several mechanisms may explain these temporal differences. Anatomically, MN is more proximal to the trigeminal root than ION; a proximal lesion may cause more complete destruction of V2 afferents, delaying regeneration and recurrence. Additionally, variations in lesion size or thermal spread between the two targets could affect axonal damage—a larger or more precise lesion at MN might disrupt more nociceptive fibers. Differential nerve regeneration capacity (greater in peripheral nerves) and the possibility of incomplete lesioning due to anatomical variations (e.g., multiple ION branches) may also contribute to earlier pain rebound in the ION‐RFT group. These findings confirm that diagnostic block‐guided selection of ION‐RFT or MN‐RFT based on pain location provides effective relief, consistent with prior reports [27, 29, 38, 39]. Thus, with careful patient selection, less invasive procedures can achieve comparable outcomes.

The 12‐month recurrence rate was 44.4% in Group I and 34.0% in Group M, with no statistically significant difference between the two groups (*p* > 0.05). Huang et al. [35] reported 1‐ and 2‐year pain relief rates of 84% and 55% following peripheral RFT, with no significant difference between initial and repeat procedures. Our recurrence rates exceeded their 1‐year rate (16%) but remained below their 2‐year rate (45%), indicating general consistency. Xu et al. [40] reported lower annual recurrence rates after percutaneous Gasserian ganglion RFT (15% and 23%), aligning with a systematic review noting higher recurrence with peripheral branch RFT [36]. Given the relatively high recurrence at 12 months, peripheral RFT may serve as a medium‐term intervention, with repeat procedures as a routine strategy upon pain recurrence.

All procedures were CT‐guided, enabling clear visualization of target foramina, fewer needle passes, and reduced injury risk. Facial numbness was the most common adverse event in both groups [41]. Masticatory muscle weakness was more frequent in Group M, likely due to proximity to motor fibers, while Group I showed slightly higher rates of puncture site swelling and transient keratitis [42], possibly related to anatomical proximity to the infraorbital artery and periocular tissues. Most adverse effects were mild and transient.

In terms of perioperative metrics, ION‐RFT was associated with significantly shorter operative time (30.020 ± 11.957 min vs. 35.660 ± 10.602 min, *p* = 0.019) and shorter hospital stay (2.130 ± 1.200 days vs. 2.790 ± 1.610 days, *p* = 0.030) compared with MN‐RFT. Since all diagnostic nerve blocks were performed in the outpatient setting prior to hospital admission, the observed difference in hospital stay reflects postoperative recovery rather than preprocedural evaluation time. Total healthcare costs did not differ significantly between the two groups (*p* = 0.694), indicating comparable cost‐effectiveness. These findings suggest that diagnostic block‐guided selection enables patients with pain confined to the infraorbital distribution to receive a less invasive procedure (ION‐RFT) with faster recovery, without incurring additional costs.

A direct MN‐RFT approach, without prior diagnostic blockade, represents a reasonable alternative strategy. Given that the ION is a distal branch of the MN, MN‐RFT would theoretically be effective in all patients with V2 TN. Such an approach would simplify the treatment pathway by eliminating sequential diagnostic blocks. However, it would expose all patients—including those with pain confined to the infraorbital distribution—to a more invasive procedure with a longer operative time. In our cohort, patients who responded to the infraorbital block (*n* = 45, approximately half of the study population) achieved comparable efficacy with ION‐RFT while benefiting from faster recovery. Thus, while direct MN‐RFT is a viable option, the diagnostic block‐guided strategy offers the advantage of patient‐centered precision.

This study has several limitations. It is a single‐center retrospective analysis with nonrandomized, sequential group allocation based on diagnostic block responses, which introduces potential selection bias. Follow‐up was limited to 12 months, leaving longer‐term outcomes uncertain. Univariate analysis precluded adjustment for confounders such as age, symptom duration, and baseline pain severity, warranting cautious interpretation. Nevertheless, all diagnostic nerve blocks were performed in the outpatient setting, so the observed differences in operative time and hospital stay reflect genuine procedural characteristics rather than preprocedural evaluation time. Future prospective studies with larger samples, multivariate analysis, and extended follow‐up are needed to confirm these findings.

## 5. Conclusion

For patients with primary V2 TN, diagnostic block‐guided selection of ION‐RFT or MN‐RFT is a safe and viable minimally invasive treatment. Both techniques offer comparable pain relief, complications, recurrence, and costs within 12 months. ION‐RFT provides significantly shorter operative time and hospital stay, reflecting faster recovery. These findings support a diagnostic block‐guided strategy that enables appropriately selected patients to receive a less invasive procedure without compromising efficacy.

## Author Contributions

Gaojian Tao designed the study and supervised the procedures. Xi Dong and Gaojian Tao performed the interventions, with Gaojian Tao providing procedural guidance. Xi Dong and Chenyu Ding prepared the figures, and Chenyu Ding created the artwork. Xi Dong and Chenyu Ding drafted the manuscript, and Gaojian Tao critically reviewed and revised the final version.

## Funding

No funding was received for this manuscript.

## Conflicts of Interest

The authors declare no conflicts of interest.

## Data Availability

The data that support the findings of this study are available on request from the corresponding author. The data are not publicly available due to privacy or ethical restrictions.
